# Recent developments and future directions in point-of-care next-generation CRISPR-based rapid diagnosis

**DOI:** 10.1007/s10238-024-01540-8

**Published:** 2025-01-09

**Authors:** Youssef M. Hassan, Ahmed S. Mohamed, Yaser M. Hassan, Wael M. El-Sayed

**Affiliations:** 1 Department of Zoology, Faculty of Science, Ain Shams University, Abbassia, Cairo 11566 Egypt; 2 Biotechnology Program, Faculty of Science, Ain Shams University, Abbassia, Cairo 11566 Egypt

**Keywords:** CRISPR-Cas systems, Nucleic acid diagnostics, Biosensing technologies, Artificial intelligence, Microfluidic platforms, Disease detection, Machine learning

## Abstract

The demand for sensitive, rapid, and affordable diagnostic techniques has surged, particularly following the COVID-19 pandemic, driving the development of CRISPR-based diagnostic tools that utilize Cas effector proteins (such as Cas9, Cas12, and Cas13) as viable alternatives to traditional nucleic acid-based detection methods. These CRISPR systems, often integrated with biosensing and amplification technologies, provide precise, rapid, and portable diagnostics, making on-site testing without the need for extensive infrastructure feasible, especially in underserved or rural areas. In contrast, traditional diagnostic methods, while still essential, are often limited by the need for costly equipment and skilled operators, restricting their accessibility. As a result, developing accessible, user-friendly solutions for at-home, field, and laboratory diagnostics has become a key focus in CRISPR diagnostic innovations. This review examines the current state of CRISPR-based diagnostics and their potential applications across a wide range of diseases, including cancers (e.g., colorectal and breast cancer), genetic disorders (e.g., sickle cell disease), and infectious diseases (e.g., tuberculosis, malaria, Zika virus, and human papillomavirus). Additionally, the integration of machine learning (ML) and artificial intelligence (AI) to enhance the accuracy, scalability, and efficiency of CRISPR diagnostics is discussed, alongside the challenges of incorporating CRISPR technologies into point-of-care settings. The review also explores the potential for these cutting-edge tools to revolutionize disease diagnosis and personalized treatment in the future, while identifying the challenges and future directions necessary to address existing gaps in CRISPR-based diagnostic research.

## Methodology

The PubMed database was used to identify relevant literature, resulting in 44 papers published between 2008 and 2024. The search focused on studies related to advancements in point-of-care (POC) next-generation CRISPR-based rapid diagnostic technologies. Keywords included “CRISPR,” “point-of-care diagnostics,” “rapid detection,” “Cas12,” “Cas13,” “CRISPR-based biosensors,” “isothermal amplification,” “lateral flow assay,” and “clinical validation.” Only articles published in English were considered. Priority was given to primary research articles for their experimental insights, with one review article included for essential background context. No clinical studies on CRISPR-based POC diagnostics with widespread deployment were identified.

## Inclusion and Exclusion Criteria

Studies were included if they:

1. Focused on the development or application of CRISPR-based diagnostic platforms,
2. Investigated POC diagnostic systems or compared them directly to traditional diagnostic methods,
3. Reported experimental data on performance metrics such as sensitivity, specificity, time-to-result, and compatibility with POC settings, and
4. Explored mechanisms to enhance detection, including integration with isothermal amplification, lateral flow assays, or fluorescence readouts.

Studies were excluded if they:

1. Did not focus on CRISPR-based diagnostics,
2. Focused solely on basic CRISPR-Cas mechanism studies without diagnostic application,
3. Lacked experimental data or performance metrics, or
4. Were unrelated to POC applications.

### Risk of bias

The risk of bias was assessed to ensure a balanced and comprehensive review. No significant bias toward positive findings was identified in the included studies. Methodological rigor was evaluated based on the use of appropriate controls, statistical analyses, and the reproducibility of results. Most studies included multiple experimental replicates and validated their findings using different sample types (e.g., synthetic, clinical). No conflicts of interest or funding-related biases were noted in the interpretation of results. Studies not published in English were excluded to maintain consistency in data interpretation and quality.

## Introduction

**Fig. 1:**
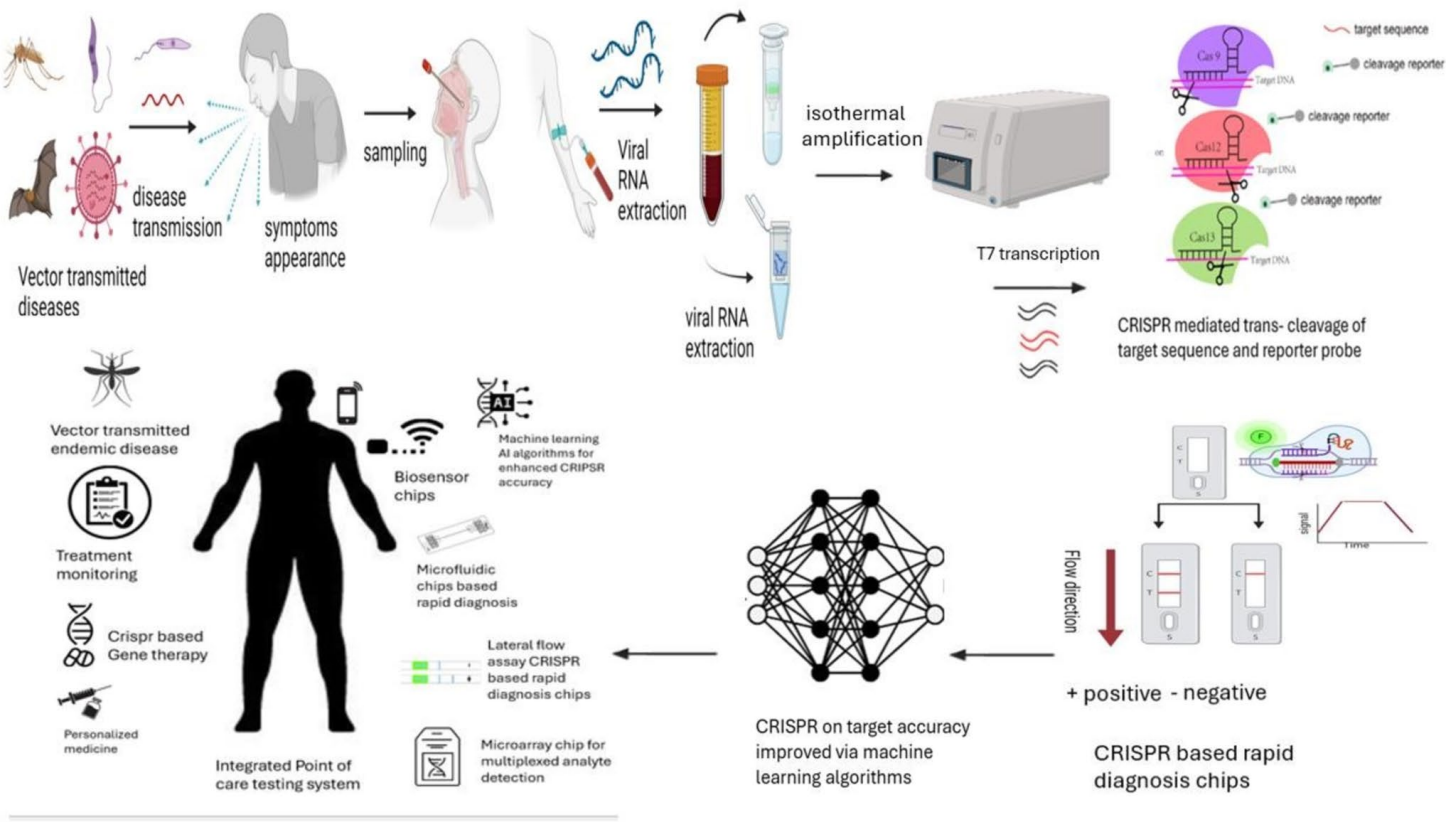
Combining AI algorithms with CRISPR-Cas diagnostics to improve point-of-care (POC) testing. The method involves several steps: nucleic acid extraction, guide RNA (gRNA) targeting disease-specific sequences, CRISPR-Cas detection, and patient sample collection. A signal indicating the presence of biomarkers is generated upon recognition of target sequences. AI algorithms enhance gRNA design, ensuring high specificity and minimal off-target effects. AI models also use data from CRISPR diagnostics to refine prediction algorithms and optimize CRISPR performance. This integrated approach enhances the accuracy and timeliness of POC tests by combining AI’s predictive power with CRISPR’s precision

The development of CRISPR-associated proteins (Cas) and clustered regularly interspaced short palindromic repeats (CRISPR) for the detection of viral nucleic acids and prognostic biomarkers represents a promising advancement in diagnostics and gene editing. Several rapid nucleic acid diagnostic kits are already available on the market. CRISPR technology, initially developed for gene editing, has been reprogrammed for diagnostic purposes due to its high specificity, sensitivity, and ease of use. The CRISPR-Cas9 and CRISPR-Cas12/13 systems have been optimized to identify cancer biomarkers with remarkable sensitivity, expanding the scope of CRISPR-based diagnostics to include both viral and nonviral biomarkers (Fig. 1). These advancements hold the potential to revolutionize cancer diagnosis by providing fast, affordable, and portable tests, particularly in resource-limited settings and rural areas [1].

**Fig. 2:**
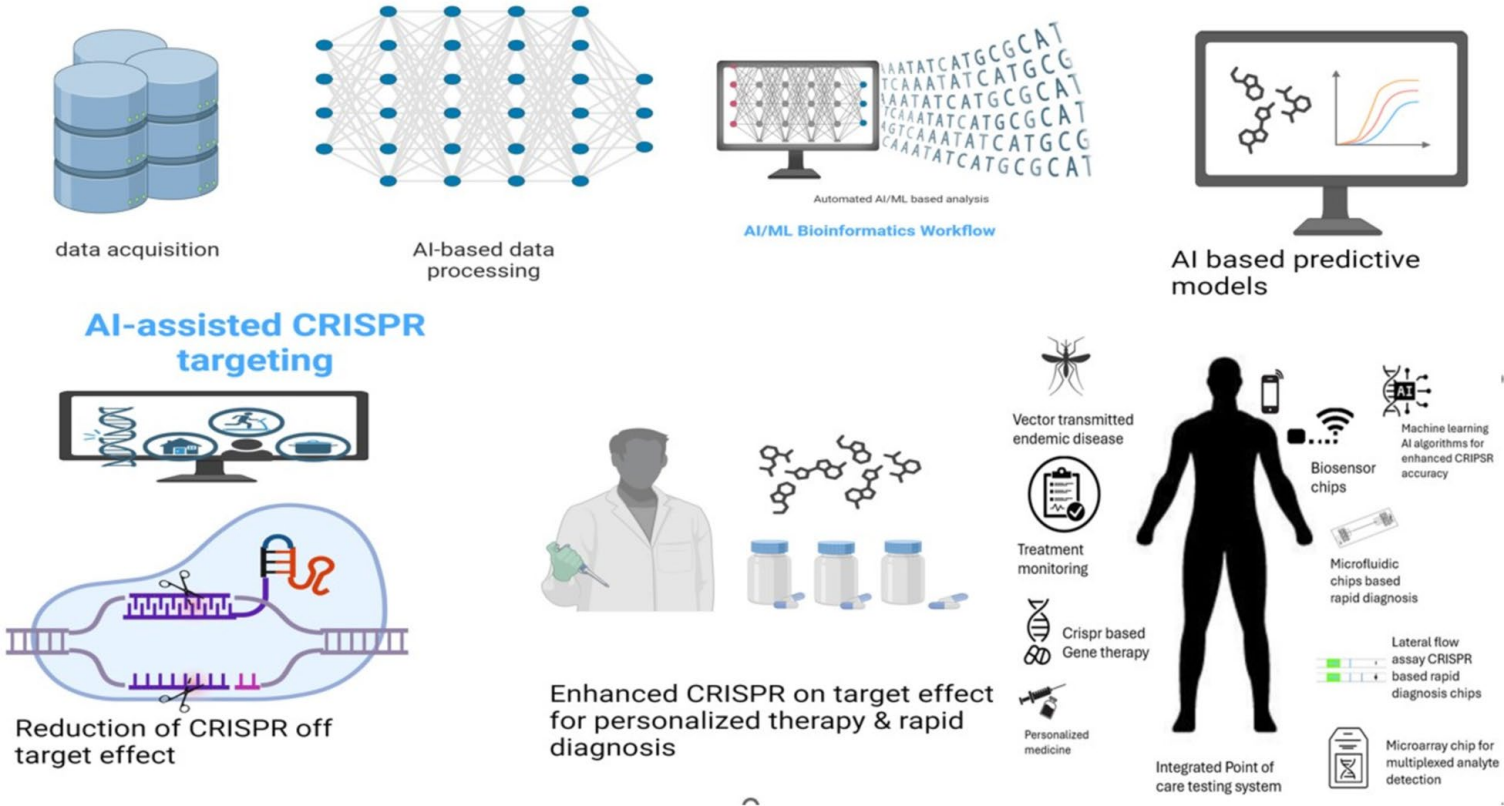
The graphic illustrates how machine learning algorithms and CRISPR technology can be combined to advance genetic research. It shows how CRISPR experiments generate genetic alteration datasets, which are then processed and analyzed using various machine learning models such as decision trees and neural networks. These models predict outcomes like gene knockout efficiency and off-target effects, with subsequent CRISPR experiments being guided by these predictions in an iterative feedback loop. The figure highlights how machine learning can enhance and accelerate CRISPR-based research and therapeutic development, with applications in gene target identification, CRISPR design optimization, and drug discovery support

However, several critical challenges remain, including the need for further validation, legal barriers, and ethical concerns surrounding genetic testing. One of the primary obstacles is improving the off-target accuracy of CRISPR systems [2, 3]. The widespread clinical adoption of CRISPR-based diagnostics hinges on overcoming these challenges, as their resolution could significantly enhance cancer prognosis and improve viral disease detection. Artificial intelligence (AI) and machine learning algorithms are increasingly being employed to refine prediction models and minimize off-target effects in CRISPR-based diagnostics (Fig. 2) [4].

The emergence of novel viral infections and cancer-related morbidities over the past two decades has threatened global healthcare, primarily due to viral mutations and the ability to recombine, leading to unique epidemiological and pathogenic traits [2, 5]. Viral diseases spread rapidly and present a significant global challenge. Outbreaks of novel viruses, such as SARS-CoV-2, H1N1 influenza, avian influenza, and dengue virus, have caused widespread economic disruption [5, 6]. While major viral diseases remain uncured, early detection and monitoring can prevent their spread. Additionally, cancer-related mortalities continue to rise, driving the demand for rapid diagnostics. As a result, new point-of-care (POC) testing kits are being developed worldwide [2].

Common techniques for multiplexed or large-scale viral detection, such as high-throughput serological assays, real-time RT-PCR, and gene sequencing, are effective but require specialized laboratories, expensive equipment, significant time, and skilled personnel. These limitations make them less suited for quick POC diagnostics. In contrast, CRISPR-based tests offer a more accessible, rapid alternative. Researchers are also developing assays that rely on isothermal amplification, such as recombinase polymerase amplification (RPA) and loop-mediated isothermal amplification (LAMP), which can be applied in various settings. However, these methods face challenges: as the reaction temperature drops (to 37°C for RPA and 65°C for LAMP), the specificity of the results decreases, complicating interpretation. In this context, the CRISPR/Cas system offers a more precise approach, capable of identifying specific sequences at lower temperatures [7].

PCR-based methods have long been regarded as the gold standard for diagnosing infectious diseases due to their high sensitivity in amplifying target DNA sequences. However, their use is limited to thorough and rapid screening because they require substantial time, expensive equipment, and specialized personnel [8, 9]. In contrast, CRISPR-Cas-based diagnostic tools, such as DETECTR and SHERLOCK, offer advantages in sensitivity and user-friendliness (Table 1). By employing RNA-guided nucleases to identify and cleave target nucleic acids, they enable rapid and precise pathogen identification without extensive sample processing [7, 10]. CRISPR-based assays are particularly valuable in resource-constrained settings as they may be less costly and more suitable for POC applications. Additionally, CRISPR-Cas systems provide specificity and the ability to distinguish between closely related genetic sequences, which are crucial for accurate diagnosis. They may also enable multiplexed detection, facilitating epidemiological surveillance and allowing the simultaneous identification of multiple pathogens in a single experiment [3, 11].

**Table 1:** Summary of CRISPR-Cas technologies used in point-of-care applications

CRISPR system	Application	Sensitivity	Specificity	Limit of detection (LOD)	Reference
Cas9	SARS-CoV-2 Detection (DETECTR system)	~ 95%	~ 98%	10 copies/µL	[12, 13]
Cas12	HPV detection (lateral flow assay)	95%	98%	10 copies/µL	[11, 14]
Cas12	*Mycobacterium tuberculosis* detection	88.3%	Specificity 94.6%	3.13 CFU/mL	[12, 15]
Cas13	Zika virus (SHERLOCK)	Attomolar	Near 100%	Attomolar	[10, 16]
Cas13	Dengue virus (SHERLOCK)	95%	98%	1 aM	[16, 17]
Cas12	SARS-CoV-2 detection (SHERLOCK)	98%	100%	10 copies/µL	[7, 12]

## CRISPR-based diagnosis for infectious diseases

Rapid diagnostic tool development has been greatly accelerated by the COVID-19 pandemic, and Cas12-based CRISPR technologies are essential for identifying SARS-CoV-2. By using Cas12’s collateral cleavage capability, these CRISPR-based assays—like the DETECTR system—are able to detect SARS-CoV-2 RNA with exceptional sensitivity and specificity. The capacity to produce results in about 30 min, as opposed to the many hours usually needed for RT-PCR, is one of the main benefits of Cas12-based CRISPR diagnostics over conventional PCR techniques. Furthermore, because CRISPR-based assays require less equipment and may be used in environments with low resources, they are ideal for point-of-care (POC) applications. By focusing on conserved sections of the SARS-CoV-2 genome, these techniques preserve excellent specificity while having sensitivity comparable to RT-PCR and detection limits as low as 10 copies/μL. These assays are also economical because they do not require costly heat cycles, which make them scalable and appropriate for large-scale testing, especially during pandemic situations. Controlling the spread of infectious diseases like COVID-19 requires quick, decentralized, and inexpensive diagnostics [12].

### CRISPR-based viral detection

Viral genomes are attractive candidates for CRISPR-based gene-editing treatments because they replicate by infecting live cells. This is particularly crucial for treating latent and chronic viral infections, such as those caused by HIV-1, HBV, and herpesviruses, as these viruses integrate their genomes into host cells, complicating identification and treatment. CRISPR systems can halt viral transcription and replication by specifically targeting and destroying viral genomes within host cells. However, practical challenges include introducing the CRISPR system into target cells, accurately targeting viral genomes despite high mutation rates (especially in HIV-1), and preventing off-target effects in the host genome while stopping viral reservoirs from establishing reinfection [18].

The discovery of RNA-guided RNA cleavage by the CRISPR-Cas13a system revolutionized viral detection. SHERLOCK and its derivatives eliminate RNA extraction, enabling applications ranging from attomolar sensitivity to single-base specificity in detecting flaviviruses like dengue and Zika [16, 17]. Cas-13a-based assays provide rapid, extraction-free detection of hemorrhagic fever viruses, including Lassa and Ebola, with good sensitivity in POC settings. Moreover, the CRISPR-Cas13a assays, including HUDSON-SHERLOCK and SHERLOCKv2, have played a role in the rapid identification of respiratory viruses, thereby enhancing pandemic control through a wide range of applications, including fluorescence readouts and lateral flow testing for SARS-CoV-2 [15, 19].

Various detection methods utilize biosensing devices of CRISPR/Cas12a technology. In the presence of target DNA, CRISPR/Cas12a cleaves ssDNA labeled with methylene blue on gold electrodes, resulting in altered current signals for detecting human papillomavirus 16 (HPV16). The reporter ssDNA is hairpin-shaped. Incorporation results show an improved sensitivity of 30 pM. Electrochemiluminescence, using ferrocene-tagged single-strand DNA and L-methionine-stabilized gold nanoclusters, yields a limit of detection (LOD) for HPV16 of 0.48 pM. Fluorescent assays created through segmentation and multiphase procedures integrate CRISPR/Cas12a with isothermal amplification, providing ultrasensitive detection of 10 copies of HPV16. Positive feedback circuits have achieved low LODs for cancer-associated DNA, reaching as low as 5 aM using exponential signal amplification. Furthermore, lateral flow strips can be combined with gold nanoparticles (AuNPs)-based colorimetry for naked-eye detection of HBV and HPV nucleic acids [14, 20, 21]. In contrast, electrochemical and fluorescent methods can be expensive in terms of instrumentation. Lateral flow strips, however, offer a portable sensing and detection alternative to traditional techniques [5, 11, 22].

The hepatitis B virus (HBV) can cause severe liver diseases; thus, accurate genotyping is crucial for determining a treatment plan. Utilizing multiple cross displacement amplification (MCDA) for rapid preamplification in conjunction with Cas12b-based detection, the CRISPR-HBV technology can detect HBV genotypes B and C with 100% specificity and a sensitivity of 10 copies per test. The real-time fluorescence test and lateral flow biosensor yield rapid results in less than an hour, including DNA extraction. CRISPR-HBV is instrumental for POC testing and is essential for determining HBV genotypes in resource-poor contexts, as validated on clinical samples [23]. This test combines MCDA preamplification, CRISPR-Cas12b, and lateral flow biosensor (LFB) output to detect HBV genotypes B and C rapidly and precisely. It differentiates between these genotypes, which is essential for customized therapy regimens due to variations in antiviral responses and clinical outcomes. Each test can detect as few as 10 copies of HBV DNA, showing high sensitivity and specificity with negligible cross-reactivity with other infections. This approach eliminates the need for expensive equipment and can be completed in 60 min, making it suitable for use in resource-constrained contexts [22].

### Isothermal PCR viral genome amplification for point-of-care testing

Polymerase chain reaction (PCR) and isothermal amplification techniques have been used to develop numerous nucleic acid detection devices [16, 24]. While PCR is the gold standard for detecting nucleic acids due to its high sensitivity and low error rate, its limitations have been mentioned before [20, 25, 26]. In contrast, isothermal amplification methods require only one temperature and do not need expensive equipment, making them more suitable for the diagnostics field, even though PCR may have issues with specificity due to nonspecific amplification [7, 27]. Diagnostic development aims to implement high-quality POC testing in resource-constrained environments. The objective is to create diagnostic tools that meet the World Health Organization’s (WHO) 2020 guidelines for sensitivity, specificity, speed, ease of use, and equipment-free delivery [13, 28]. However, accuracy and cost have trade-offs; rapid antigen testing is less expensive than more advanced laboratory-based nucleic acid testing, which tends to be more sensitive and specific [18, 29].

### Multiplexed viral CRISPR-Cas diagnostic assay

**Fig. 3:**
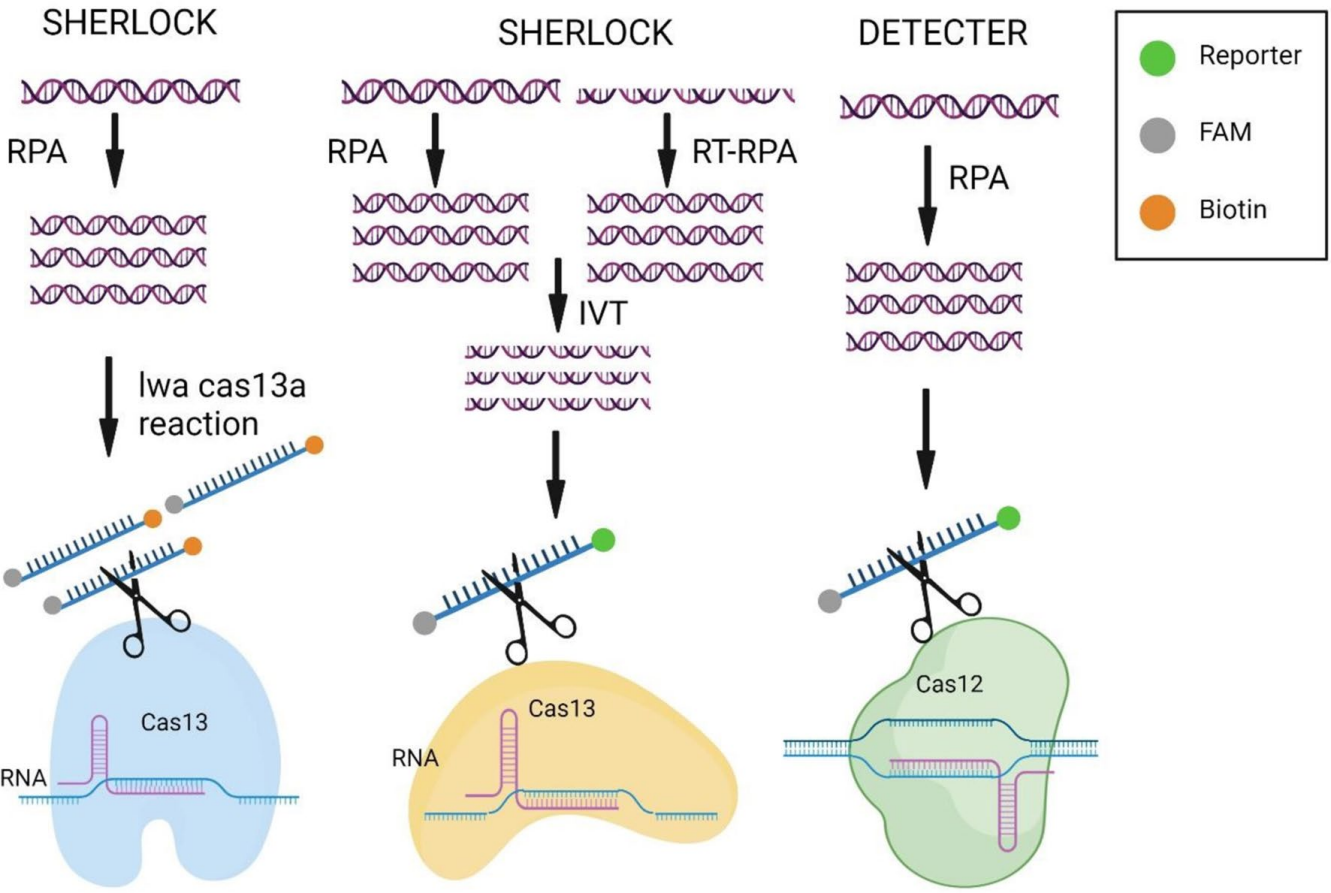
This figure illustrates CRISPR-Cas technologies for nucleic acid detection using SHERLOCKv2, SHERLOCK, and DETECTR assays. The Cas nuclease is inactive in the absence of its nucleic acid target. When the nuclease binds to its guide crRNA and recognizes a similar target (RNA for Cas13a or ssDNA/dsDNA for Cas12a), it becomes activated and cleaves off-target nucleic acids. The collateral nuclease activity is enhanced through the use of reporter probes, which consist of a fluorophore attached to a quencher via a short oligonucleotide. The figure is divided into three parts: **Left**: SHERLOCKv2, which allows direct detection of viral infections in body fluids using RNA or DNA as input. **Middle**: SHERLOCK, which amplifies nucleic acids from clinical samples using recombinase polymerase amplification (RPA). **Right**: DETECTR, which employs a similar nucleic acid detection method, starting with RT-RPA or RPA, followed by detection with T7 RNA polymerase, Cas13, a target-specific crRNA, and an RNA reporter that fluoresces upon cleavage

The need for a diagnostic tool to identify several high-risk human papillomavirus (hrHPV) types linked to cervical cancer led to the development of the CRISPR-Cas multiplexed diagnostic assay (CRISPRD) [30, 31]. The assay detects HPV16 and HPV18 in a single-pot reaction with 100% specificity and a sensitivity of 10 copies, including an internal control using thermostable AapCas12b, TccCas13a, and HheCas13a nucleases in conjunction with isothermal amplification. This cutting-edge approach shows promise for wider applications in multiplex detection of additional nucleic acid biomarkers, in addition to providing a quick solution for hrHPV detection (Fig. 3). Due to its versatility, CRISPRD has the potential to enhance large-scale hrHPV screening programs, aligning with international health objectives aimed at reducing the incidence of cervical cancer through early detection [11].

### CRISPR-based bacterial diagnosis

*Mycobacterium tuberculosis* (MTB) spreads through aerosols causing tuberculosis (TB) which poses a serious threat to global public health. Developing practical, economical, accurate, and efficient tests for MTB is essential for managing and preventing tuberculosis. Improving the sensitivity of MTB detection is crucial for prompt TB diagnosis and treatment [12, 32]. Traditional smear acid-fast staining requires a large bacterial load, which limits sensitivity and increases the chance of missed diagnoses. Drug-resistant tuberculosis (TB) remains challenging to diagnose quickly due to lengthy culture procedures. A recent study developed a rapid method for MTB detection by combining CRISPR/Cas technology with isothermal amplification, achieving a detection limit of 3.13 CFU/mL. With a sensitivity of 88.3% and a specificity of 94.6%, TB-CRISPR identified 504 probable TB patients within just 1.5 h, significantly reducing detection time compared to methods like Xpert MTB/RIF and culture [12, 15].

The 1.5-h timeframe refers to the entire sample-to-result process, encompassing sample preparation, isothermal amplification, and detection. In comparison, the Xpert MTB/RIF assay typically requires approximately 2 h for detection, while traditional culture methods take 1 to 8 weeks, depending on bacterial growth rates and laboratory conditions [12, 15]. These advancements highlight the potential of CRISPR-based diagnostics in resource-constrained settings, where timely diagnosis is critical for early treatment and improved outcomes.

### CRISPR-based diagnosis of neglected tropical diseases (NTDs)

**Fig. 4:**
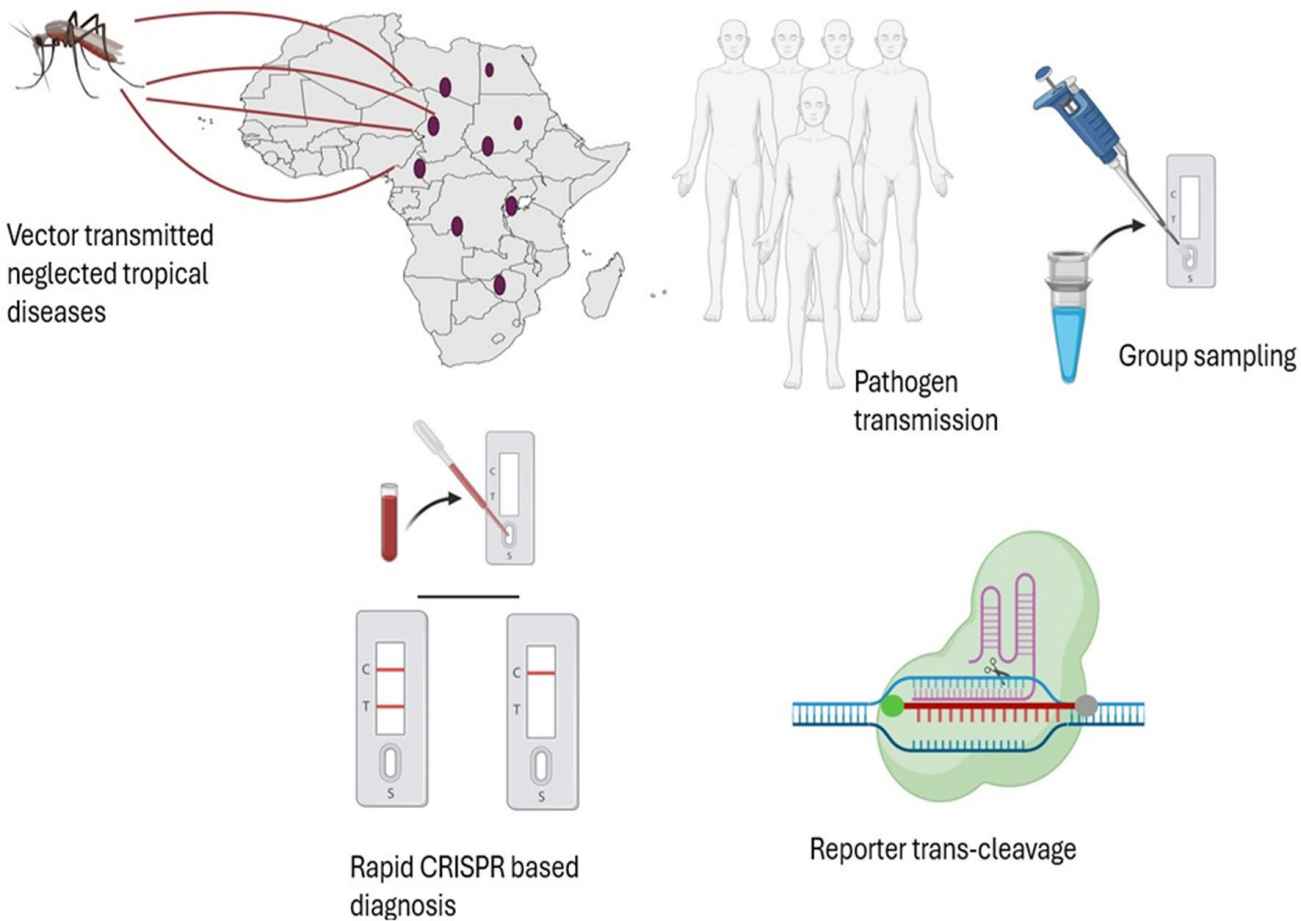
CRISPR and AI-based integrated point-of-care system for rapid malaria diagnosis. This figure illustrates an integrated point-of-care (POC) system for the rapid diagnosis of malaria. AI algorithms enhance the accuracy of the system. First, nucleic acids are extracted from patient samples, after which the CRISPR-Cas system targets DNA regions associated with *Plasmodium falciparum*. The CRISPR-Cas12a enzyme recognizes the target sequence and uses fluorophore-quencher probes or FAM-Biotin probes in lateral flow assays, to generate signals with the help of specific guide RNAs (gRNAs). AI algorithms are used to design the gRNAs, ensuring low off-target effects and high specificity. The performance of the CRISPR system is further improved, and the prediction algorithms are continuously refined, by feeding diagnostic data into AI models. This integration combines the rapid and accurate detection capabilities of CRISPR with AI’s predictive power

CRISPR technology has transformed the identification and treatment of malaria through precisely modified genes. Sensitive diagnostic techniques like SHERLOCK and DETECTR can accurately identify malaria DNA or RNA patterns using CRISPR enzymes such as Cas12 and Cas13 (Fig. 4). These techniques provide straightforward readouts, enabling accurate and timely results via lateral flow measurements. Improvements to CRISPR-based diagnostics include their incorporation into portable devices for wider application. Despite current barriers to rapid field adoption, SHERLOCK technology holds promise for drug resistance surveillance, research, and malaria diagnosis [20]. Single-nucleotide variant (SNV) detection methods extend beyond diagnosing infectious diseases; they allow for pathogen genotyping at the nucleotide level, facilitating tracking of malaria treatment resistance without the need for PCR or sequencing [9, 13]. Pilot projects utilizing SHERLOCK to identify *Plasmodium falciparum* in mosquitoes indicate potential for wider application in low-transmission malaria regions. Further developments in Cas effectors, workflow optimization, and POC capabilities are expected to enhance CRISPR diagnostics for clinical and surveillance applications, particularly in resource-limited settings [33, 34].

## CRISPR-based tumor biomarkers detection

Circulating tumor DNA (ctDNA), produced from necrotic and apoptotic cancer cells, is present in the bloodstream and carries mutations from both primary and metastatic cancers. The detection of ctDNA is essential for cancer diagnosis. Determining epidermal growth factor receptor (EGFR) gene alterations facilitates assessments of cancer progression and the effectiveness of therapies. crRNAs that specifically target EGFR790 and EGFR858 to detect mutant EGFR using CRISPR/Cas12a were developed. Another study employed AuNPs-modified ssDNAs in a CRISPR/Cas12a-based approach to identify breast cancer gene-1 [12]. At a concentration of 0.34 fM LOD, target ctDNA activation cleaved unpaired ssDNA, leading to linked AuNP dissociation, which changed the solution’s color and enhanced the metal-induced fluorescence signal, despite the rare presence of the required 5′TTTN-3′ PAM sequence in genomic DNA, indicating great sensitivity [35, 36].

Colorectal cancer (CRC) is becoming a significant global health concern due to its rising incidence and mortality rates, particularly in wealthier nations. Most current monitoring strategies rely on complex molecular methods like RT-qPCR and invasive procedures such as colonoscopies. Recent studies have shown that extracellular vesicles (EVs), specifically exosomes, play a critical role in intercellular communication. They carry miRNA, which is associated with cancer growth and angiogenesis. CEx-miRNA shows potential as a biomarker for colorectal cancer prognosis and diagnosis [37]. Compared to existing screening methods, new molecular techniques like CRISPR-Dx technologies offer early detection capabilities at a reduced cost, making them potentially beneficial. CRISPR/Cas13-based technologies can improve treatment outcomes by identifying CRC risk early and enabling POC diagnosis due to their sensitivity, scalability, and ease of use. Future research avenues may enhance CRISPR/Cas13 for miRNA detection, including direct CRISPR/Cas13-miRNA detection or SHERLOCK-based assays. These advancements may facilitate the identification of CEx-miRNA from less invasive materials, such as blood, using specific miRNA panels (miR-126, miR-1290) associated with early-stage colorectal cancer [32, 38].

### CRISPR-based miRNA detection

Numerous nucleic acid amplification methods integrate with CRISPR/Cas systems to enhance the sensitivity and specificity of miRNA detection. One such integration is PCR with CRISPR/Cas12a, which requires heat cycling but increases sensitivity to 9 fM for miRNAs like let-7a. Isothermal techniques such as CRISPR/Cas13a hyperbranching rolling circle amplification can identify miR-17 at 200 aM with high selectivity and fewer steps. CRISPR/Cas13a direct recognition techniques enable precise cleavage of miR-17 RNA, resulting in an LOD of 4.5 amol and the ability to detect single-nucleotide changes. Cascade amplification techniques enhance the activity of CRISPR/Cas12a, achieving clinical-level sensitivity through DNAzyme-mediated RNA transcript cleavage [17, 39, 40]. The combination of catalytic hairpin assembly (CHA) with CRISPR/Cas12a allows enzyme-free amplification, enabling sensitive and portable miRNA detection in clinical settings. These advancements demonstrate the versatility of CRISPR/Cas systems for nucleic acid detection, boosting their sensitivity and specificity, and making it possible to identify a wider array of miRNA biomarkers [29, 36, 41].

## Development of CRISPR-based protein detection method

Research is ongoing into nucleic acid sensing systems, such as CRISPR-Cas, for detecting proteins and small molecules critical for POC diagnostics. Adapting these systems to target entities beyond nucleic acids, such as proteins and small molecules, presents ongoing challenges. For instance, a Cas12a-crRNA system targets protein-bound aptamers in an electrochemical CRISPR sensing platform, establishing a dose-dependent relationship for signal detection with an LOD of 0.2 nM for transforming growth factor β1 [42]. Similarly, small molecules like ATP bound by a DNA aptamer can stimulate the expression of Cas12a-crRNA, resulting in fluorescence emissions that can be measured with a standard fluorimeter, achieving a LOD of 0.75 μM. These methods demonstrate the versatility of CRISPR-Cas systems for biosensing applications beyond nucleic acids. Additionally, transcription factors specific to bacteria can be utilized to identify non-nucleic acid targets, modulating protein function through structural changes and activating Cas12a’s trans-cleavage activity, which can be detected via fluorescence. This technology successfully identified uric acid and p-hydroxybenzoic acid in blood samples within 15 to 25 min. Although the technique has only been demonstrated in vitro, intracellular biosensing may be feasible due to the integration of CRISPR into cellular genetics and allosteric regulatory networks [18, 43].

## CRISPR-based point-of-care diagnosis of clinical pathogens

CRISPR technology has fundamentally transformed molecular diagnostics, offering rapid and reliable detection of various pathogens, including those responsible for oral infections. In oral health, CRISPR-based techniques such as CRISPR-Cas12a quickly identify bacterial infections like *Streptococcus mutans* in saliva samples. When a specific crRNA guide binds to bacterial DNA, the Cas12a enzyme exhibits collateral cleavage activity, producing a fluorescent signal that indicates the pathogen’s presence. Furthermore, CRISPR-Cas13a systems facilitate the rapid identification of viral infections including herpes simplex virus (HSV) and human papillomavirus (HPV) [6]. CRISPR technology also aids in diagnosing fungal infections, particularly those caused by *Candida albicans*, through precise targeting of fungal DNA sequences. The ease of obtaining oral cavity samples enhances the efficacy of this approach for prompt clinical diagnosis. Additionally, CRISPR enables the simultaneous identification of multiple diseases through multiplex tests, shedding light on the diverse bacteria in the oral cavity for more detailed investigations. The development of CRISPR-based portable POC diagnostic instruments represents a significant advancement, allowing for quick and accurate identification of oral infections at dental offices or even at home—essential in remote or resource-constrained areas [24]. CRISPR-based detection enhances patient outcomes and facilitates early diagnosis in oral healthcare [31].

## Technologies development for point-of-care testing applications

### Microfluidic chips for point-of-care (POC) testing

Lab-on-a-chip devices, commonly referred to as microfluidic chips, are compact systems that enable the manipulation and analysis of very small fluid volumes, typically in the microliter or nanoliter range. By integrating multiple laboratory operations onto a single chip, these devices shorten processing times, reduce reagent use, facilitate high-throughput screening, and enable prompt diagnoses. Microfluidic technology has significantly advanced POC testing, offering affordable, portable, and effective diagnostic tools for various settings, including remote or resource-constrained environments [17] (Fig. 2). Recent advancements in microfluidic chip technology have broadened applications for proof-of-concept testing. The combination of CRISPR-based detection systems with microfluidic chips has led to the creation of highly sensitive and tailored diagnostic tools. For instance, integrating CRISPR-Cas12 and CRISPR-Cas13 systems into microfluidic devices enables precise and rapid nucleic acid detection from pathogens without the need for sample preparation [42].

#### Paper-based microfluidics

Paper-based microfluidic devices have emerged as user-friendly proof-of-concept testing platforms, utilizing capillary action to transfer fluids without extra pumps or power sources. Recent improvements in the sensitivity and specificity of these devices have enabled them to detect various analytes, including pathogens, environmental pollutants, and biomarkers. Enhanced microfluidic chips, facilitated by advances in 3D printing and microfabrication, are now achievable, allowing for greater control over semiconductor design and integration of multiple capabilities, thus improving overall device performance. POC diagnostics, drug testing, and personalized medicine are increasingly utilizing 3D-printed microfluidic chips.

#### Digital microfluidics

Digital microfluidics, involving the manipulation of discrete droplets on a hydrophobic surface using electric fields, opens avenues for automated and high-throughput testing. Recent advancements have made digital microfluidics more scalable and reliable, making it a viable approach for various diagnostic applications. However, several challenges remain before microfluidic chips can be widely applied for proof-of-concept testing. Accurate sample processing and handling are major issues, and the complexity of preparing multiple microfluidic devices may limit their use in resource-constrained contexts [13, 40]. On-chip processing advancements and integrated sample preparation are necessary to address these barriers. While microfluidic devices have made significant strides in sensitivity and specificity, further developments are needed for accurately detecting low-abundance analytes. Additionally, the cost of developing and scaling microfluidic devices poses a significant challenge. Although paper-based and 3D-printed chips are less expensive, ensuring consistent quality and performance at scale is essential for broader application. For successful POC testing, microfluidic devices must be user-friendly enough for nonexperts to operate. Robust designs and intuitive interfaces are essential for effective use in various circumstances, including by healthcare professionals with limited technical experience. Standardizing microfluidic devices and obtaining regulatory approval for clinical use can also be challenging and time-consuming, necessitating precise standards and processes for development and validation to ensure safety and efficacy in clinical settings.

### The next generation of CRISPR technology aided by artificial intelligence algorithms

Recent advancements have highlighted the complementary roles of AI and CRISPR in diagnostic applications, pushing the boundaries of CRISPR-based precision medicine, particularly in the era of big data analysis. Artificial intelligence enhances CRISPR’s gene-editing potential by expediting data processing and optimizing target recognition [14]. This synergy optimizes diagnostic methods, enabling faster and more efficient disease detection, which is crucial for timely intervention and epidemiological surveillance [17, 33]. The healthcare sector stands to undergo a radical transformation as tailored medicine advances and becomes more prevalent in POC settings [19]. One novel strategy involves using CRISPR technology for the sensitive detection of viruses and food contaminants in the headspace of packaged food, which may help protect consumer health [28, 38, 40]. CRISPR-based intelligent packaging technology is expected to provide real-time surveillance of food contaminants in the food industry. Additionally, exploring how deep learning can enhance food quality inspection may lead to a safer and more secure environment, advancing global food safety [16].

**Fig. 5:**
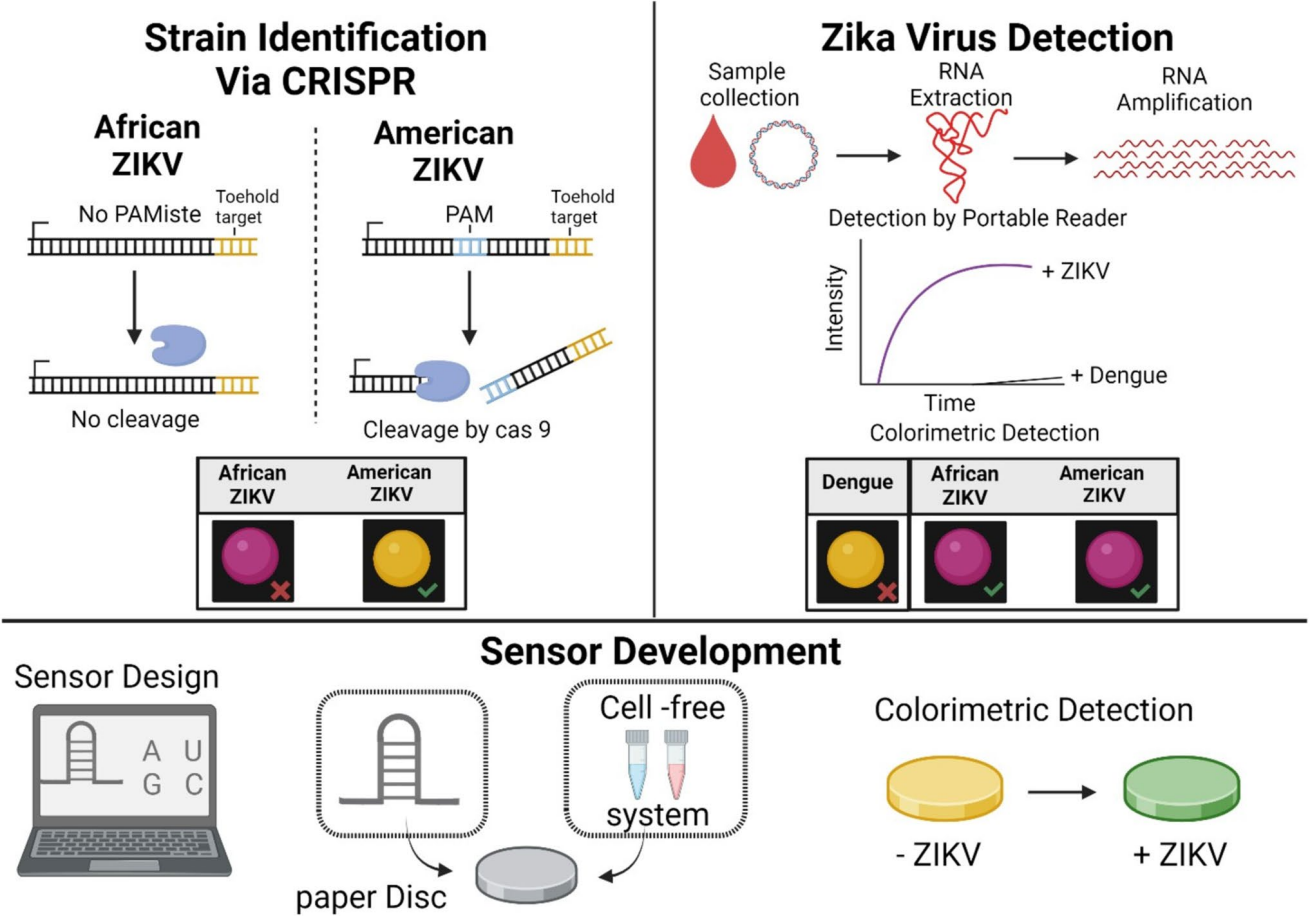
Strain distinction at single-base resolution is achievable with the NASBA-CRISPR cleavage (NASBACC) approach, as demonstrated by genotyping of the Zika virus. The activation of a toehold switch sensor is influenced by a synthetic trigger sequence, which is unique to strain variations after NASBA amplification of RNA. PAM sites specific to a strain generate either full-length or shortened RNA, affecting sensor activation. Sensor 32B can distinguish between dengue RNA and Zika strains but cannot differentiate between American and African strains of the Zika virus. To overcome this limitation, NASBACC exploits a single-base SNP that results in a PAM site exclusive to the American strain. This allows Cas9 to cleave the RNA of the American strain, generating shortened RNA that does not activate the sensor, while the RNA of the African strain remains intact and triggers the sensor. The NASBACC framework provides accurate genotypic data and has potential practical applications, as Cas9 is compatible with lyophilization

Recent studies illustrate the rapid development of CRISPR-Cas technology in biosensing, particularly in food safety applications. These biosensors utilize Cas proteins (Cas12a, Cas13a, and Cas14a) for highly sensitive detection of pathogens like *Vibrio parahaemolyticus* and *Listeria monocytogenes* using methods such as electrochemical sensors and nucleic acid chips. CRISPR-based systems like SHERLOCK and DETECTR facilitate rapid detection of bacteria and viruses, including *E. coli* and *Salmonella*, as well as viruses like dengue and Zika, without requiring specialized tools [2, 4] (Fig. 5). These technologies enhance food security by addressing critical challenges like allergies, antibiotic resistance, and viral contamination. There is growing interest in researching smart biosensor systems, which encompass smart biosensors, implants, and prosthetics. These systems can assist healthcare professionals in monitoring and predicting diseases for early intervention, utilizing nanochips, nanosensors, and nanorobots for detection and drug tracking [38].

#### Internet of things (IoT) technology

**Fig. 6:**
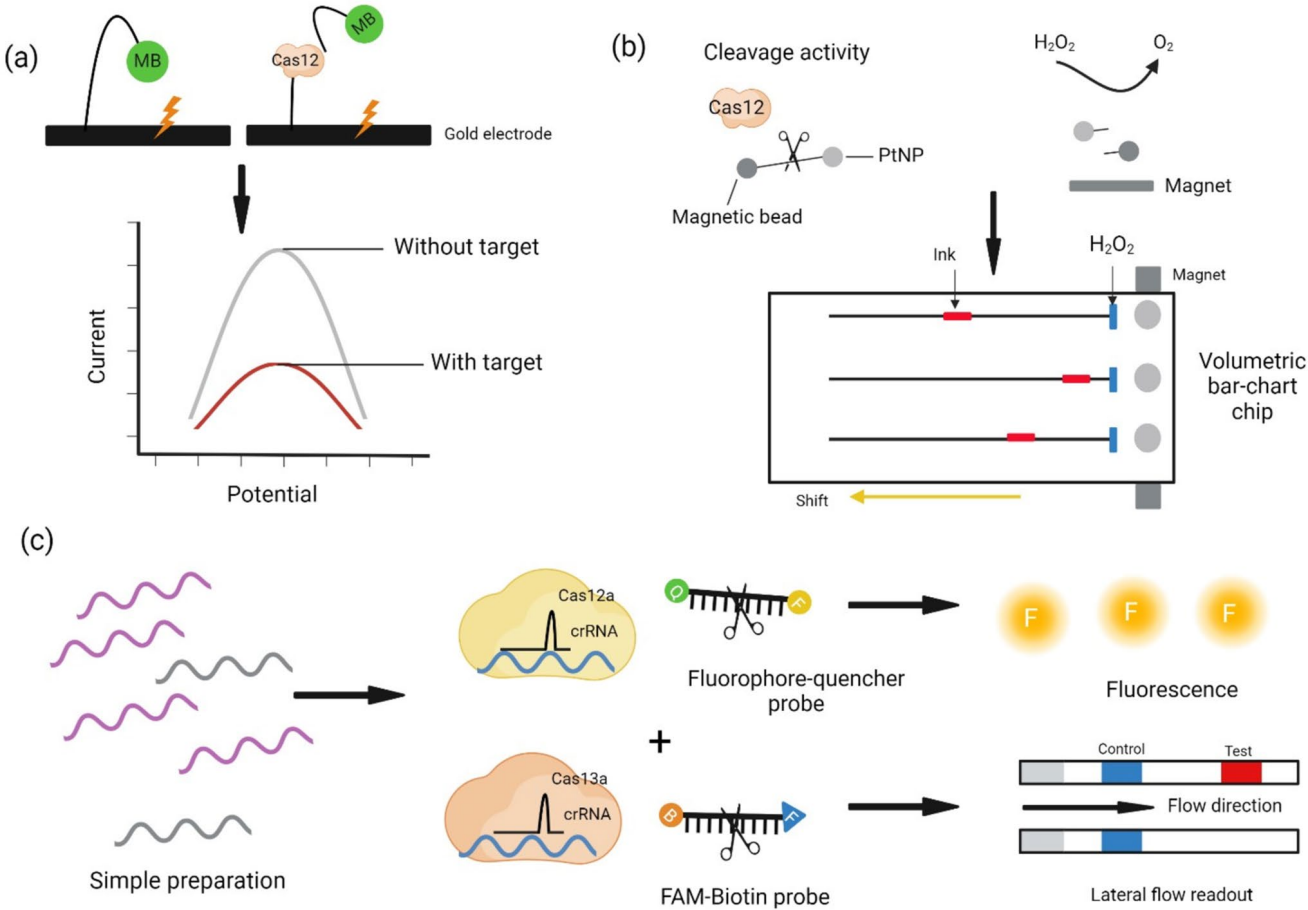
AI algorithms enhance CRISPR-mediated point-of-care diagnosis. This figure illustrates the process of CRISPR-mediated point-of-care diagnostics. The first step involves nucleic acid extraction from patient samples. Next, disease biomarkers are targeted using CRISPR-Cas systems, such as Cas12 and Cas13a, which are guided by RNA sequences. The detection process generates signals through fluorescence or lateral flow readouts. Diagrammatic depiction of CRISPR-Cas-based diagnostic systems employing Cas12 and Cas13: **a** The figure shows CRISPR-Cas-based detection techniques using a lateral flow readout (lower panel) or a fluorescent probe (upper panel). Variants of CRISPR-Cas cleave single-stranded DNA (ssDNA) or RNA (ssRNA) that is bound to fluorophore-quencher or FAM-biotin reporters, after targeting specific pathogenic DNA or RNA sequences. FAM-tagged molecules are visible on paper strips, while fluorescence devices detect and quantify the emitted fluorescent signals. **b** This panel shows a methylene blue (MB) probe integrated into an electrochemical biosensor (E-CRISPR). The electric current decreases as a result of the enzyme cleaving the ssDNA linker of the MB electrochemical tag when CRISPR-Cas12 identifies the target sequence. **c** A magnet-assisted volumetric bar-chart chip (MAV-chip) combined with CRISPR-Cas12a and platinum nanoparticles (PtNP) is shown in this panel. When target DNA is present, Cas12 cleaves the neighboring ssDNA reporter that is connected to PtNP and a magnetic bead, in addition to the target sequence. PtNP facilitates the conversion of H₂O₂ to O₂. The quantity of target DNA is measured by the change in ink on the chip, caused by the liberation of O₂ gas

Technological advancements in networking, data analysis, and artificial intelligence have led to the emergence of the Internet of Things (IoT), Internet of Nano Things (IoNT), Industrial Internet of Things (IIoT), and Internet of Medical Things (IoMT). These technologies are transforming healthcare by enhancing diagnosis, patient information management, real-time monitoring, and emergency management through wireless communication between medical devices and healthcare personnel. Nanotechnology plays a crucial role in developing nanoscale devices, including nanochips, nanosensors, and nano-routers, which track environmental allergens and infections while monitoring patient health using implanted nanosensors. CRISPR-based biosensors have significantly advanced molecular diagnosis (Fig. 6) by providing a reliable, user-friendly, and efficient method for diagnosing diseases through the identification of DNA or RNA sequences using CRISPR/Cas systems. However, challenges remain in automating these systems for POC applications, including limitations in signal translation, data management, and microprocessor manufacturing [27, 43].

#### Biosensing technologies utilizing artificial intelligence

The integration of artificial intelligence (AI) and machine learning (ML) has transformed biosensing technologies, providing innovative approaches for monitoring and diagnosing diseases. These advancements address various medical challenges, including cancer, infectious diseases, genetic disorders, and cardiovascular conditions. Data mining enhances our understanding, diagnosis, and prediction of diseases, making machine learning algorithms increasingly vital in clinical and pathological diagnosis.

Machine learning algorithms are generally categorized into three types: reinforcement learning, unsupervised learning, and supervised learning [16, 42]. Supervised machine learning (SML) is commonly employed in tasks like image classification and pattern recognition, utilizing techniques such as neural networks (NNs), random forests, support vector machines, and decision trees. Unsupervised learning employs methods like rule mining and clustering to predict outcomes from unlabeled data by identifying learned patterns. Reinforcement learning focuses on teaching software to optimize behavior in specific situations, though it is less frequently applied in this context.

The convergence of AI with biosensors has led to the creation of smart biosensors. For example, the XPRIZE DeepQ Tricorder biosensor can diagnose 12 different conditions while monitoring vital signs in real-time. AI has been effectively utilized in various biosensing applications, such as deep learning for lung cancer diagnosis. CRISPR/Cas9 and dCas9-based biosensors demonstrate high flexibility and sensitivity in identifying genetic markers and infections [36]. These methods utilize single-guide RNAs to target and cut DNA sequences, leveraging the precision of the CRISPR/Cas9 system. Enhanced detection sensitivity allows for distinguishing single-nucleotide polymorphisms through techniques like NASBA and CAS-EXPAR [16, 17, 25, 29]. Moreover, dCas9-based biosensors combined with nanomaterials such as graphene or microring resonators provide highly sensitive, amplification-free detection. Applications range from detecting viral strains and tick-borne diseases to diagnosing genetic defects and pathogens, highlighting CRISPR technology’s potential for precise and rapid diagnostics. CRISPR-based diagnostics are particularly promising for POC and home-use applications due to their sensitivity and specificity [34].

#### A recording mechanism based on DNA

The development of CRISPR-based DNA recording devices offers a promising approach for tracking cellular activity. These devices provide a solid platform for biological research, capable of identifying and recording a wide range of signals, converting them into alterations in DNA, and storing data for future analysis [35, 38]. By capturing diverse signals and translating them into genetic changes, CRISPR-based DNA recording systems establish a practical method for monitoring cellular processes [42].

## Challenges facing CRISPR-based point-of-care (POC) diagnostics (Table 2)

**Table 2:** Summary of challenges facing CRISPR-based diagnosis technology in point-of-care testing

Technology/Approach	Key achievements	Limitations	References
SHERLOCK (Cas13-based)	Attomolar sensitivity; rapid detection of RNA viruses like Zika and dengue; multiplex capability	Requires preamplification; challenges with direct application in resource-limited settings	[10, 14, 25]
DETECTR (Cas12-based)	High specificity for SARS-CoV-2 RNA; rapid results (~ 30 min); suitable for POC applications	Cold-chain reagent logistics; limited scalability for field deployment	[7, 12, 27]
Lateral flow assays (LFAs)	Portable, equipment-free detection of nucleic acids for diseases like HPV and HBV	Lower sensitivity compared to fluorescence methods; requires optimization for multiplexed detection	[11, 18, 20]
CRISPR-Cas multiplex assays	Simultaneous detection of multiple pathogens; application in high-risk HPV diagnostics	Complex gRNA design; needs cost-effective solutions for wide-scale adoption	[30, 31]
Microfluidic chips with CRISPR	Compact, high-precision diagnostics for respiratory viruses; integration of sample-to-result workflows	Production cost and complexity; limited validation in resource-constrained environments	[21, 42]

CRISPR technology offers immense potential in revolutionizing diagnostics, yet several critical challenges hinder its widespread adoption, particularly in POC settings. A primary obstacle is the complexity of sample processing, which often requires labor-intensive and specialized procedures unsuitable for rapid testing environments. Current nucleic acid extraction and processing techniques demand advanced equipment and skilled personnel, limiting their accessibility in resource-constrained areas. Simplifying these processes through strategies such as direct lysis methods, one-step reaction chemistries, and integrated automation can greatly enhance usability in POC applications [20, 44].

Logistical and storage requirements also present significant hurdles. Many reagents used in CRISPR diagnostics necessitate transport and storage at very low temperatures, complicating implementation in remote or underserved regions. Advances in stabilization methods, like lyophilized reagents that remain effective at room temperature, could eliminate reliance on cold-chain logistics, making CRISPR diagnostics more practical and scalable [20].

For CRISPR-based diagnostics to achieve regulatory approval and widespread adoption, they must meet rigorous standards for standardization, robustness, and reliability. Factors like temperature, humidity, and user expertise can impact diagnostic performance, making it imperative to validate these systems across diverse scenarios and environments [11, 23, 30, 31]. Furthermore, ensuring cost-effectiveness is a pressing challenge, as current production processes and materials may drive up costs. Developing scalable production methods without compromising diagnostic accuracy will be essential for making these technologies accessible globally.

AI integration into CRISPR diagnostics introduces significant advantages in addressing many of these challenges. AI enhances the accuracy, speed, and scalability of CRISPR systems by expediting data analysis and optimizing target recognition. For instance, machine learning models can identify specific genetic sequences associated with diseases, predict the most effective guide RNAs (gRNAs), and minimize off-target effects [12, 35]. These advancements optimize both the activity and specificity of CRISPR-based diagnostic systems, facilitating their application in personalized medicine by tailoring tools to detect patient-specific mutations or infections [12, 35].

In large-scale settings, AI-driven automation increases throughput and simplifies data interpretation, enabling CRISPR diagnostics to handle extensive sample sizes with minimal human intervention [19, 20, 33]. This capability is crucial for epidemiological research, as predictive modeling supported by AI can forecast disease outbreaks and monitor the spread of infectious diseases, enhancing public health responses [12].

Another promising area of innovation is in CRISPR-based biosensors, particularly for applications like detecting pathogens in food safety or environmental monitoring. Emerging technologies, such as CRISPR electrochemical biosensors, provide quick, accurate, and equipment-free detection solutions [12]. These advancements could transform diagnostics in resource-constrained settings, although regulatory and ethical challenges remain. Evolving frameworks will need to address concerns surrounding genetic testing and editing, ensuring the responsible application of CRISPR technology [23, 30].

By addressing these challenges, streamlining sample processing, improving logistics, ensuring reliability, and leveraging AI, the full potential of CRISPR-based POC diagnostics can be realized, making them transformative tools for global healthcare.

## Summary

CRISPR-based technologies have revolutionized molecular diagnostics, enabling the accurate and rapid detection of diseases, particularly in point-of-care (POC) settings. This study highlights the sensitivity, flexibility, and versatility of CRISPR-based diagnostic tools, emphasizing their broad applications in identifying a range of pathological conditions, including cancer, genetic disorders, and infectious diseases. Real-time, on-site diagnostics using affordable, user-friendly technologies are now possible, thanks to techniques such as SHERLOCK, DETECTR, and emerging CRISPR-Cas12/13-based approaches, which have demonstrated remarkable efficacy in detecting specific DNA and RNA sequences.

The integration of CRISPR into diagnostic platforms, including electrochemical biosensors, fluorescence-based assays, and lateral flow assays (LFAs), has enabled rapid and highly sensitive testing procedures. CRISPR-based diagnostics also offer the potential for multiplexed detection, allowing the simultaneous monitoring of multiple biomarkers for more comprehensive disease profiling. Innovations in paper-based diagnostics and microfluidic devices are further improving the affordability and accessibility of CRISPR-based technologies, particularly in resource-limited settings.

Advancements in integrating CRISPR with artificial intelligence (AI) and machine learning (ML) algorithms are enhancing diagnostic accuracy, speed, and scalability. AI can optimize real-time data processing and guide the selection of guide RNAs (gRNAs) for targeting specific sequences. This synergy between CRISPR and AI is expected to transform personalized medicine by enabling customized therapies and improving the treatment of complex diseases.

Additionally, studies exploring CRISPR for protein and small molecule detection have broadened its potential beyond nucleic acid diagnostics. CRISPR-Cas12a systems, in particular, show promise for high-sensitivity protein detection, and future advancements in CRISPR-based sensors are expected to yield amplification-free, real-time diagnostic tools for a wide range of diseases.

## Future prospectives

There are numerous opportunities for CRISPR-based diagnostics to advance in the future. Key areas of focus include:

### Integration with AI and ML

The combination of CRISPR and artificial intelligence (AI) holds enormous potential to enhance diagnostic processes. Machine learning can analyze the large datasets generated by CRISPR diagnostics, identifying new biomarkers and optimizing the selection of guide RNAs (gRNAs). AI can also accelerate data processing, helping to develop predictive models for treatment responses and disease outbreaks.

### Miniaturization and portability

Advancements in lab-on-a-chip technology, paper-based devices, and microfluidic chips are making CRISPR-based diagnostics more accessible and portable. These miniaturized devices are expected to broaden the application of CRISPR diagnostics in non-laboratory settings, particularly in underserved or rural areas, by offering real-time, affordable disease diagnoses.

### Multiplexed detection

One promising area of research is the ability to simultaneously detect multiple biomarkers for different diseases in a single test. When combined with cutting-edge microfluidic technology and AI, CRISPR’s multiplexing capability could enable highly efficient multi-disease diagnostics, saving time and costs in both clinical practice and disease surveillance.

### Point-of-care (POC) integration

The integration of CRISPR-based diagnostics into point-of-care (POC) systems is becoming increasingly feasible as these technologies evolve. Simplifying and automating diagnostic processes will enable faster, more accurate diagnoses with minimal need for complex infrastructure or specialized personnel—particularly in resource-limited settings.

### Therapeutic applications

Beyond diagnostics, CRISPR’s potential in therapeutic applications—such as gene editing to treat genetic disorders and precision medicine—is growing. The development of CRISPR-based diagnostic tools that are directly linked to individualized treatment plans is advancing the field of precision medicine.

### Global health impact

CRISPR-based diagnostics could play a pivotal role in addressing global health challenges, especially in detecting and monitoring emerging infections and neglected tropical diseases (NTDs). With further advancements, CRISPR-based assays could be used in low-resource environments, offering rapid, accurate, and cost-effective diagnostic options that could significantly improve patient outcomes worldwide.

## Data Availability

No datasets were generated or analyzed during the current study.

## Abbreviations

CRISPR: Clustered regularly interspaced short palindromic repeats
Cas: Crisper associated
RPA: Recombinase polymerase amplification
LAMP: Loop-mediated isothermal amplification
SHERLOCK: Specific High-sensitivity Enzymatic Reporter unLOCKing
NABSA: Nucleic acid-based sequence amplification
SML: Supervised machine learning
NNs: Neural networks
SVM: Support vector machine
ML: Machine learning
EXPAR: Exponential amplification reaction
IoT: Internet of Things
IoNT: Internet of Nano Things
IIoT: Industrial Internet of Things
IoMT: Internet of Medical Things
